# Risk and Timing of Noncardiac Surgery After Transcatheter Aortic Valve Implantation

**DOI:** 10.1001/jamanetworkopen.2022.20689

**Published:** 2022-07-07

**Authors:** Taishi Okuno, Caglayan Demirel, Daijiro Tomii, Gabor Erdoes, Dik Heg, Jonas Lanz, Fabien Praz, Rainer Zbinden, David Reineke, Lorenz Räber, Stefan Stortecky, Stephan Windecker, Thomas Pilgrim

**Affiliations:** 1 Department of Cardiology, Inselspital, University of Bern, Bern, Switzerland; 2 Department of Anesthesiology and Pain Medicine, Inselspital, University of Bern, Bern, Switzerland; 3 Clinical Trials Unit, University of Bern, Bern, Switzerland; 4 Department of Cardiovascular Surgery, Inselspital, University of Bern, Bern, Switzerland

## Abstract

**Importance:**

Noncardiac surgery after transcatheter aortic valve implantation (TAVI) is a clinical challenge with concerns about safety and optimal management.

**Objectives:**

To evaluate perioperative risk of adverse events associated with noncardiac surgery after TAVI by timing of surgery, type of surgery, and TAVI valve performance.

**Design, Setting, and Participants:**

This cohort study was conducted using data from a prospective TAVI registry of patients at the tertiary care University Hospital in Bern, Switzerland. All patients undergoing noncardiac surgery after TAVI were identified. Data were analyzed from November through December 2021.

**Exposures:**

Timing, clinical urgency, and risk category of noncardiac surgery were assessed among patients who had undergone TAVI and subsequent noncardiac surgery.

**Main Outcomes and Measures:**

A composite of death, stroke, myocardial infarction, and major or life-threatening bleeding within 30 days after noncardiac surgery.

**Results:**

Among 2238 patients undergoing TAVI between 2013 and 2020, 300 patients (mean [SD] age, 81.8 [6.6] years; 144 [48.0%] women) underwent elective (160 patients) or urgent (140 patients) noncardiac surgery after TAVI and were included in the analysis. Of these individuals, 63 patients (21.0%) had noncardiac surgery within 30 days of TAVI. Procedures were categorized into low-risk (21 patients), intermediate-risk (190 patients), and high-risk (89 patients) surgery. Composite end points occurred within 30 days of surgery among 58 patients (Kaplan-Meier estimate, 19.7%; 95% CI, 15.6%-24.7%). There were no significant differences in baseline demographics between patients with the 30-day composite end point and 242 patients without this end point, including mean (SD) age (81.3 [7.1] years vs 81.9 [6.5] years; *P* = .28) and sex (25 [43.1%] women vs 119 [49.2%] women; *P* = .37). Timing (ie, ≤30 days from TAVI to noncardiac surgery), urgency, and risk category of surgery were not associated with increased risk of the end point. Moderate or severe prosthesis-patient mismatch (adjusted hazard ratio [aHR], 2.33; 95% CI, 1.37-3.95; *P* = .002) and moderate or severe paravalvular regurgitation (aHR, 3.61; 95% CI 1.25-10.41; *P* = .02) were independently associated with increased risk of the end point.

**Conclusions and Relevance:**

These findings suggest that noncardiac surgery may be performed early after successful TAVI. Suboptimal device performance, such as prosthesis-patient mismatch and paravalvular regurgitation, was associated with increased risk of adverse outcomes after noncardiac surgery.

## Key Points

**Question:**

What factors are associated with increased perioperative risk of noncardiac surgery adverse outcomes after transcatheter aortic valve implantation (TAVI)?

**Findings:**

In this cohort study of 300 patients undergoing noncardiac surgery after TAVI, timing, urgency, and risk category of noncardiac surgery were not associated with increased risk of perioperative adverse events. Suboptimal TAVI valve performance (ie, prosthesis-patient mismatch and paravalvular regurgitation) was associated with increased risk of adverse clinical events within 30 days of noncardiac surgery.

**Meaning:**

These findings suggest that noncardiac surgery may be performed early after successful TAVI; suboptimal device performance was associated with increased risk of adverse outcomes after noncardiac surgery.

## Teaser

This cohort study investigates the association of surgery and valve factors with adverse outcomes of noncardiac surgery following transcatheter aortic valve implantation.

## Introduction

Noncardiac surgery elicits cardiac stress and hemodynamic alterations due to tissue injury, fluid shifts, and effects of vasoactive agents, potentially causing adverse cardiovascular events. The presence of severe aortic stenosis (AS) is associated with substantial increases in perioperative risk associated with noncardiac surgery^1,2,3,4,5,6^ and mandates aortic valve replacement (AVR) prior to surgery according to guidelines from the European Society of Cardiology and European Association for Cardio-Thoracic Surgery (ESC/EACTS) and American College of Cardiology and American Heart Association (AHA/ACC).^7,8^ Transcatheter aortic valve implantation (TAVI) offers a minimally invasive approach associated with a short reconvalescence period minimizing delay between valve replacement and noncardiac surgery.^9,10^ Furthermore, patients may require elective or urgent noncardiac surgery months or years after TAVI.

As a result, noncardiac surgery in patients with previous TAVI is a challenging scenario that is encountered in clinical practice with increasing frequency.^11,12^ TAVI and noncardiac surgery have the potential to mutually impact outcomes at the valve site, as well as at the surgical intervention level. On 1 side, suboptimal hemodynamic outcome after TAVI may be associated with a residual risk after noncardiac surgery, and antithrombotic treatment is associated with increased hazard of bleeding. Conversely, noncardiac surgery after TAVI creates a prothrombotic milieu associated with unknown outcomes for the valve prosthesis and increased risk of infection and endocarditis.^13^

Available evidence on the safety of noncardiac surgery after TAVI is limited to small case series and does not provide guidance on the timing of noncardiac surgery after valve replacement or factors associated with procedural risk.^14,15^ We aimed to evaluate perioperative risk of adverse events associated with noncardiac surgery following TAVI by timing of surgery, type of surgery, and TAVI valve performance.

## Methods

The Bern TAVI registry was approved by the Bern cantonal ethics committee, and all patients provided written informed consent for participation. The original registry's ethics committee approval and satisfaction of informed consent requirement extend to this study. This cohort study was conducted in compliance with the Declaration of Helsinki and conforms to the Strengthening the Reporting of Observational Studies in Epidemiology (STROBE) reporting guideline.

### Study Design and Population

The Bern TAVI registry is a prospective registry enrolling consecutive patients undergoing TAVI at Bern University Hospital, which forms part of the nationwide Swiss TAVI registry (NCT01368250). For this study, noncardiac surgery after TAVI was captured using hospital discharge summaries, documentation from referring physicians, and telephone interviews for patients registered between January 2013 and July 2020. The study cohort for this analysis comprised all patients who underwent noncardiac surgery after TAVI. Procedures for TAVI-associated complications during the same hospitalization were not considered as noncardiac surgery.

### Data Collection and Outcome Measures

A web-based database with standardized case report forms was used for prospective data collection (baseline clinical, procedural, and follow-up data). Baseline clinical data were updated to the time of noncardiac surgery using clinical follow-up data recorded in the registry, documentation from referring physicians, and hospital discharge summaries. For noncardiac surgery, the date of surgery, elective vs urgent or emergent indication for surgery, and type of surgery were collected. Type of surgery was categorized as low-, intermediate-, or high-risk procedure according to ESC and European Society of Anesthesiology noncardiac surgery (ESC/ESA-NCS) guidelines.^16,17^ The first noncardiac surgery was considered when a patient underwent surgery multiple times.

Standardized transthoracic echocardiography was performed by a board-certified cardiologist and an echocardiography specialist with a Philips iE33 machine (Phillips) at day 2 or 3 after TAVI and before hospital discharge at the latest. Paravalvular regurgitation (PVR) was graded as none or trace, mild, moderate, or severe according to a 3-class grading scheme using a multiparametric and integrative approach described by updated Valve Academic Research Consortium (VARC) criteria.^18^ Prosthesis-patient mismatch (PPM) was categorized based on prosthetic effective orifice area indexed to body surface area as severe (≤0.65 cm^2^/m^2^) or moderate (>0.65 to 0.85 cm^2^/m^2^) in the population without obesity and as severe (≤0.55 cm^2^/m^2^) or moderate (>0.55 to 0.70 cm^2^/m^2^) in the population with obesity (body mass index [calculated as weight in kilograms divided by height in meters squared] ≥ 30).

In the Bern TAVI registry, clinical follow-up was performed at 30 days, 1 year, and 5 years after TAVI. Clinical event data were obtained in a standardized manner, blinded for patient details, and adjudicated based on original source documents by a dedicated clinical event committee using updated VARC criteria.^18^ An independent clinical trials unit is responsible for central data monitoring to verify completeness and accuracy of data and independent statistical analysis.

The primary outcome measure in this study was a composite end point of all-cause death, stroke (disabling and nondisabling), myocardial infarction, and major or life-threatening bleeding (end points as defined by Valve Academic Research Consortium-2) at 30 days after noncardiac surgery. Additional follow-up and event adjudication were needed for 8 patients whose regular follow-up had not reached 30 days after noncardiac surgery.

### Statistical Analysis

Categorical variables are reported as frequencies and percentages. Continuous variables are presented as mean values with SDs. Univariable and multivariable Cox proportional hazard models were used to calculate hazard ratios (HRs), 95% CIs, and *P* values. Variables with *P* < .10 in univariable analysis were entered in the multivariable model. Missing data were handled using multiple imputation by chained equations for the multivariable model. The imputation model included all variables shown in the footnote of the multivariable model. We imputed 20 data sets, and estimates obtained were combined according to Rubin rules. Actuarial time-to-event curves were depicted using the Kaplan-Meier method. Spline estimations were used to model the association between time from TAVR to noncardiac surgery and the 30-day composite end point, with knots at 30, 60, and 365 days. In all time-to-event analyses, data for a patient were censored at the time of the first event for that patient. All *P* values were 2-sided, and *P* < .05 was considered significant for all tests. All statistical analyses were performed with Stata statistical software version 15.1 (StataCorp). Data were analyzed from November through December 2021.

## Results

**Figure 1.**
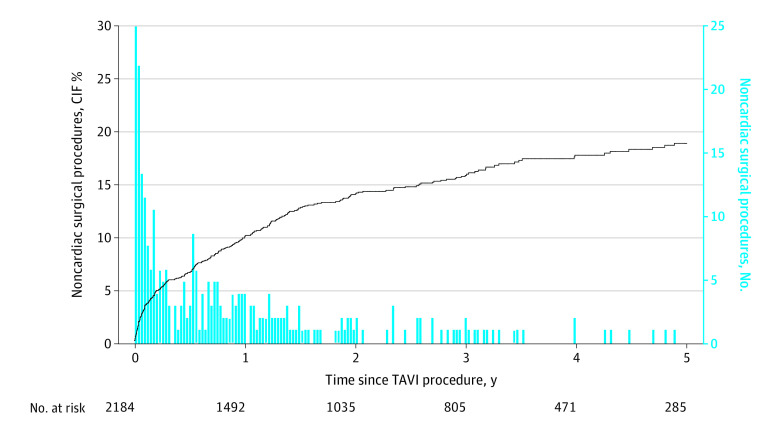
Cumulative Frequency of Noncardiac Surgery After Transcatheter Aortic Valve Implantation (TAVI) CIF indicates cumulative incidence function.

Among 2238 patients undergoing TAVI between January 2013 and July 2020, 300 patients (13.4%; mean [SD] age, 81.8 [6.6] years; 144 [48.0%] women) underwent elective (160 patients) or urgent or emergent (140 patients) noncardiac surgery until September 2021 and were included in the analysis. Of these individuals, 63 patients (21.0%) had noncardiac surgery within 30 days of TAVI, 75 patients (25.0%) between 31 days and 180 days of TAVI, 69 patients (23.0%) between 181 days and 365 days of TAVI, and 93 patients (31.0%) later than 1 year after TAVI (Figure 1). Among all patients with TAVI, 207 patients (9.2%) underwent noncardiac surgery within 1 year after TAVI. Patients were categorized into ESC/ESA-NCS low-risk (21 patients [7.0%]), intermediate-risk (190 patients [63.3%]), and high-risk (89 patients [29.7%]) surgery groups. Details of procedures are provided in Table 1. Neurological or orthopedic major surgery (98 patients [32.7%]) was the most common procedure, followed by superficial (47 patients [15.7%]) and intraperitoneal (41 patients [13.7%]) surgery. Patient demographic, comorbidity, medication, TAVI, and surgery data are shown in Table 2.

**Table 1.** Patients by Surgical Risk Estimate and Type of Surgery or Intervention

Surgery risk and type	Patients, No. (%)
High risk^a^	
Aortic or major vascular surgery	2 (0.7)
Open lower limb revascularization, amputation, or thromboembolectomy	12 (4.0)
Repair of perforated bowel	2 (0.7)
Adrenal resection	1 (0.3)
Pneumonectomy	3 (1.0)
Pulmonary or liver transplant	1 (0.3)
Intermediate risk^a^	
Intraperitoneal surgery (splenectomy, hiatal hernia repair, or cholecystectomy)	41 (13.7)
Carotid symptomatic surgery (CEA or CAS)	1 (0.3)
Peripheral artery angioplasty	31 (10.3)
Endovascular aneurysm repair	1 (0.3)
Head or neck surgery	8 (2.7)
Neurological or orthopedic surgery, major (hip and spine)	98 (32.7)
Urological or gynecological, major surgery	4 (1.3)
Intrathoracic, non-major surgery	6 (2.0)
Low risk^a^	
Superficial surgery	47 (15.7)
Breast surgery	7 (2.3)
Eye surgery	3 (1.0)
Carotid asymptomatic surgery (CEA or CAS)	2 (0.7)
Minor gynecologic, orthopedic, or urological surgery	30 (10.0)

Abbreviations: CAS, carotid artery stenting; CEA, carotid endarterectomy.

^a^ Current guidelines from the European Society of Cardiology and European Society of Anaesthesiology on noncardiac surgery divide surgical procedures into those associated with low (<1%), intermediate (1%-5%), and high (>5%) risk of major adverse cardiac events (cardiac death and myocardial infarction).

**Table 2.** Patient and Surgery Characteristics

Characteristic	Patients, No. (%)			*P* value^a^
	Total (N = 300)	With 30-d composite end point (n = 58)	Without 30-d composite end point (n = 242)	
Patient^b^				
Age, mean (SD), y	81.8 (6.6)	81.3 (7.1)	81.9 (6.5)	.28
Women	144 (48.0)	25 (43.1)	119 (49.2)	.37
Concomitant disease				
Hypertension	256 (85.3)	52 (89.7)	204 (84.3)	.29
Diabetes	92 (30.7)	23 (39.7)	69 (28.5)	.09
Dyslipidemia	203 (67.7)	40 (68.9)	163 (67.4)	.77
Coronary artery disease	182 (61.0)	39 (67.2)	143 (59.1)	.22
Kidney failure (eGFR < 60 mL/min/1.73m^2^)	101 (33.7)	16 (27.5)	85 (35.1)	.33
Peripheral artery disease	39 (13.0)	11 (18.9)	28 (11.6)	.17
COPD	38 (12.7)	8 (13.8)	30 (12.4)	.86
Patient history				
CVE	52 (17.3)	9 (15.5)	43 (17.8)	.67
MI	42 (14.0)	10 (17.2)	32 (13.2)	.44
PCI	94 (31.3)	24 (41.4)	70 (28.9)	.07
Medication				
Aspirin	196 (67.9)	37 (66.7)	159 (68.2)	.69
P2Y12 inhibitor	125 (43.1)	24 (43.9)	101 (43.3)	.86
VKA	53 (18.3)	15 (26.3)	38 (16.3)	.09
DOAC	65 (22.4)	12 (21.1)	53 (22.7)	.94
TAVI procedure				
Indication				
Native aortic valve stenosis	282 (94.0)	53 (91.4)	229 (94.6)	.33
Native aortic valve regurgitation	5 (1.7)	2 (3.4)	3 (1.2)	.25
Failed bioprosthesis	13 (4.3)	3 (5.2)	10 (4.1)	.68
Transfemoral access	292 (97.3)	56 (96.6)	236 (97.5)	.70
Valve type^c^				
Balloon expandable	152 (50.8)	28 (48.3)	124 (51.5)	.90
Self-expanding	121 (40.5)	24 (41.4)	97 (40.2)	
Mechanically expandable	26 (8.7)	6 (10.3)	20 (8.3)	
Valve generation^d^				
Early generation	51 (17.1)	6 (10.3)	45 (18.7)	.13
Newer generation	248 (82.9)	52 (89.7)	196 (81.3)	
Valve size				
≤23 mm or Symetis S	46 (15.4)	10 (17.2)	36 (14.9)	.64
>23 mm or Symetis M, L	253 (84.6)	48 (82.8)	205 (85.1)	
Echocardiographic outcome after TAVI				
Indexed EOA, mean (SD), cm^2^/m^2^	0.93 (0.27)	0.86 (0.24)	0.95 (0.28)	.048
Mean transprosthetic gradient, mean (SD), mm Hg	8.51 (4.30)	8.67 (4.99)	8.47 (4.12)	.79
PPM				
Moderate	60 (24.4)	21 (43.8)	39 (19.7)	.002
Severe	22 (8.9)	5 (10.4)	17 (8.6)	
Moderate or severe	82 (33.3)	26 (54.2)	56 (28.3)	.001
Moderate or severe PVR	6 (2.0)	4 (6.9)	2 (0.8)	.003
Surgery				
Time from TAVI to noncardiac surgery				
Mean (SD), d	332 (391)	436 (459)	307 (370)	.014
≥30 d	63 (21.0)	12 (20.7)	51 (21.1)	.91
Urgent surgery	140 (46.7)	34 (58.6)	106 (43.8)	.05
ESC/ESA-NCS risk category				
High	89 (29.7)	18 (31.0)	71 (29.3)	.80
Intermediate	190 (63.3)	35 (60.3)	155 (64.0)	
Low	21 (7.0)	5 (8.6)	16 (6.6)	

Abbreviations: COPD, chronic obstructive pulmonary disease; CVE, cerebrovascular event; DOAC, direct oral anticoagulant; eGFR, estimated glomerular filtration rate; EOA, effective orifice area; ESC/ESA-NCS, European Society of Cardiology and European Society of Anesthesiology noncardiac surgery; MI, myocardial infarction; PCI, percutaneous coronary intervention; PPM, prosthesis-patient mismatch; PVR, paravalvular regurgitation; TAVI, transcatheter aortic valve implantation; VKA, vitamin-K antagonist.

^a^ *P* values are obtained from univariable Cox regressions (dependent variable is time to 30-day composite end point after noncardiac surgery).

^b^ Patient characteristics are at time of noncardiac surgery.

^c^ Balloon-expandable valves were Sapien XT, 3, and 3 Ultra (Edwards Lifesciences). Self-expanding valves were CoreValve and Evolut R and Pro (Medtronic), Acurate neo (Boston Scientific), and Portico (Abbott). Mechanically expanding valves were Lotus (Boston Scientific).

^d^ Early generation valves were Sapien XT (Edwards Lifesciences) and CoreValve. Newer-generation valves were others.

Clinical follow-up at 30 days after noncardiac surgery was complete in all patients. Composite end points occurred within 30 days of noncardiac surgery among 58 patients (Kaplan-Meier estimate, 19.7%; 95% CI, 15.6%-24.7%), including all-cause death among 28 patients (Kaplan-Meier estimate, 9.6%; 95% CI, 95% CI, 6.7%-13.5%), stroke among 3 patients (Kaplan-Meier estimate, 1.1%; 95% CI, 0.3%-3.2%), myocardial infarction in 1 patient (Kaplan-Meier estimate, 0.4%; 95% CI, 0.1%-2.6%), and major or life-threatening bleeding among 33 patients (Kaplan-Meier estimate, 11.3%; 95% CI, 8.2%-15.6%). There were no significant differences in baseline comorbidities or demographics between patients with adverse events after noncardiac surgery and 242 patients without these events, including mean (SD) age (81.3 [7.1] years vs 81.9 [6.5] years; *P* = .28) and sex (25 [43.1%] women vs 119 [49.2%] women; *P* = .37). TAVI procedural characteristics were comparable between groups; however, patients with a 30-day composite end point had a smaller mean (SD) indexed effective orifice area (0.86 [0.24] cm^2^/m^2^ vs 0.95 [0.28] cm^2^/m^2^; *P* = .048) and were more likely to have moderate or severe PPM (26 of 48 patients with PPM data [54.2%] vs 56 of 198 patients with PPM data [28.3%]; *P* = .001) and moderate or severe PVR (4 patients [6.9%] vs 2 patients [.8%]; *P* = .003) compared with patients without the 30-day composite end point. The mean (SD) time to noncardiac surgery was significantly longer (436 [459] days vs 307 [370] days; *P* = .01) in patients with vs those without the 30-day composite end point, while the prevalence of ESC/ESA-NCS risk categories for noncardiac surgery and rate of early noncardiac surgery within 30 days of TAVI were similar between groups (Table 2).

**Figure 2.**
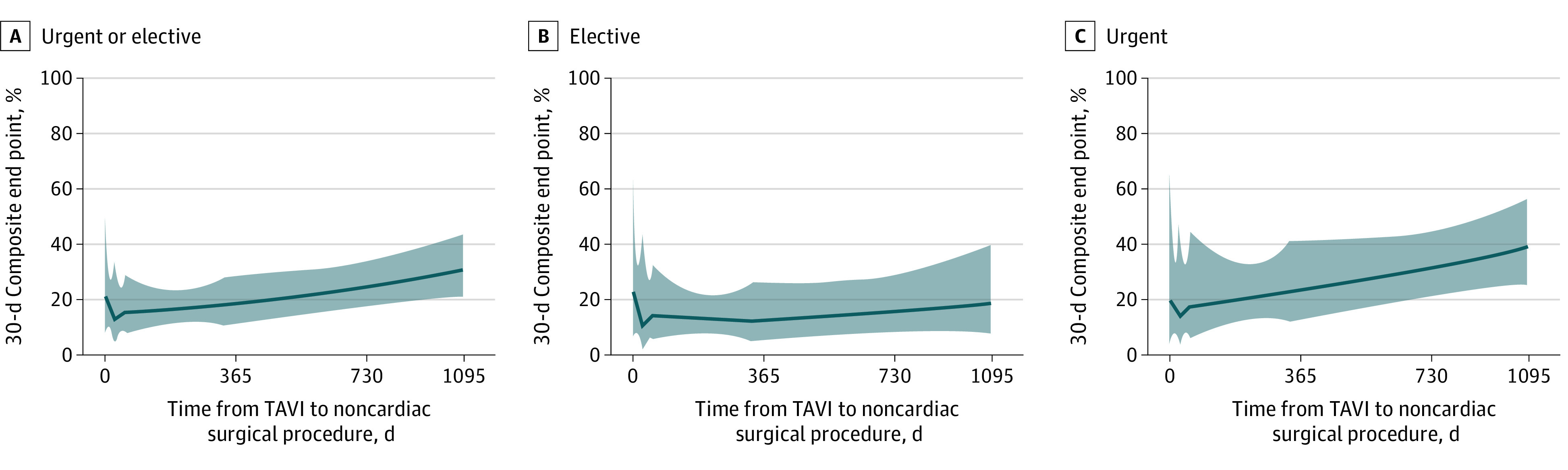
Association Between Noncardiac Surgery Timing and 30-d Composite End Point Rate of patients with 30-day composite end point is shown by days after transcatheter aortic valve implantation (TAVI). Shaded areas indicate 95% CIs; solid lines, estimated rate. Estimates were adjusted for age.

Associations between timing of noncardiac surgery in comparison with TAVI and occurrence of the composite end point at 30 days, adjusting for age, are shown in Figure 2. In multivariable analysis, moderate or severe PPM (adjusted hazard ratio [aHR], 2.33; 95% CI, 1.37-3.95; *P* = .002) and moderate or severe PVR (aHR, 3.61; 95% CI 1.25-10.41; *P* = .02) were independently associated with increased risk of the 30-day composite end point after noncardiac surgery, while urgent or emergent surgery and ESC/ESA-NCS high-risk or intermediate-risk surgery were not (Table 3).

**Table 3.** Multivariable Analysis for 30-d Composite End Point^a^

Variable	aHR (95%CI)	*P* value
History of PCI	1.61 (0.95-2.72)	.08
Moderate or severe PPM	2.33 (1.37-3.95)	.002
Moderate or severe PVR	3.61 (1.25-10.41)	.02
Urgent surgery	1.60 (0.94-2.73)	.08
Diabetes	1.33 (0.78-2.29)	.30

Abbreviations: aHR, adjusted hazard ratio; PCI, percutaneous coronary intervention; PPM, prosthesis-patient mismatch; PVR, paravalvular regurgitation.

^a^ Multiple imputation using all patients; imputed were indexed effective orifice area, mean transprosthetic gradient, device size, kidney failure, and PPM. Used to model this imputation were nonmissing variables: moderate or severe PVR, noncardiac surgery, surgery for transcatheter aortic valve implantation complications, sex, age, hypertension, diabetes, dyslipidemia, coronary artery disease, chronic obstructive pulmonary disease, history of cerebrovascular event, history of myocardial infarction, history of percutaneous coronary intervention, peripheral arterial disease, previous pacemaker, native aortic valve stenosis, native aortic valve regurgitation, failed bioprosthesis, femoral access, valve generation, and device success. Rubin's rule compiled from 20 imputed data sets.

## Discussion

The salient findings of this registry-based cohort study evaluating perioperative risk associated with noncardiac surgery after TAVI can be summarized as follows: Nearly 1 of 10 patients underwent noncardiac surgery within 1 year after TAVI, and nearly one-fifth of noncardiac operations were performed within 30 days after TAVI. Risk of the composite end point within 30 days of noncardiac surgery was approximately 20%, with a 30-day mortality rate of nearly 10% in this population of older patients who underwent TAVI. Time from TAVI to surgery, urgency of surgery, and risk category of surgery were not associated with increased risk of perioperative adverse events. Suboptimal TAVI outcome (specifically, relevant PPM and PVR) was associated with increased risk of adverse clinical events within 30 days of noncardiac surgery.

Severe AS limits blood flow from the left ventricle into the aorta. Chronic pressure overload caused by the fixed obstruction results in compensatory concentric left ventricle hypertrophy, which reduces myocardial compliance, as well as coronary flow reserve. Consequently, patients with severe AS are at increased risk of hemodynamic alterations associated with surgical and anesthetic stress of noncardiac surgery.^6^ Furthermore, patients with severe AS may have an increased risk of perioperative bleeding associated with acquired von Willebrand^19^ and Heyde syndrome.^20^ Indeed, observational studies have consistently shown an increased risk of adverse cardiovascular events associated with noncardiac surgery in patients with AS,^1,2,3,4,5,6^ leading to the guideline recommendation of prophylactic AVR before elective noncardiac surgery.^7,8^

Two retrospective studies^21,22^ have explored the association of prior or prophylactic AVR with noncardiac surgery outcomes. In a single-center study^21^ among 491 patients with severe AS, patients with AVR prior to noncardiac surgery had decreased risk of major adverse cardiovascular events within 30 days of noncardiac surgery compared with patients without AVR (5.4% vs 20.5%; *P* < .001). Similarly, in a Japanese multicenter registry study^22^ among 348 patients with severe AS, 30-day mortality after noncardiac surgery was lower in patients who had undergone AVR before noncardiac surgery compared with those with untreated severe AS (0% vs 4.3%; *P* = .008).

There are 2 shortcomings of prophylactic AVR before noncardiac surgery. First, the accumulation of 2 successive major surgeries represents a major stress and is associated with prolonged reconvalescence. Second, prophylactic AVR is potentially associated with delayed noncardiac surgery owing to the time required for recovery from open-heart surgery. A delay in the treatment of cancer is associated with worse patient prognosis.^23^ Aside from cancer surgery, delay of elective surgery may be associated with lower quality of life for patients, as well as increased risk of infectious complications and mortality in case of in-hospital delay.^24,25^ In this regard, TAVI, which is associated with faster recovery^10^ and shorter length of hospital stay than surgical AVR,^9^ may play an important role.^8^ In a recent case series of patients who had undergone prophylactic TAVI, major noncardiac surgery was safely performed at a median time of 37 days after TAVI.^14^

In our study, there was no association between time from TAVI to noncardiac surgery and adverse clinical events after noncardiac surgery. Even noncardiac surgery within 30 days of TAVI was not associated with increased risk of 30-day adverse clinical events compared with noncardiac surgery at later periods after TAVI. This finding provides evidence in support of a strategy of prophylactic TAVI promptly followed by noncardiac surgery for patients with severe AS requiring noncardiac surgery.

Another important finding of this analysis is that suboptimal hemodynamic outcomes, specifically moderate or severe PPM and moderate or severe PVR, were independently associated with a more than 2-fold and nearly 4-fold increased risk of 30-day adverse clinical events after noncardiac surgery, respectively. Although there are conflicting data regarding the association of PPM with mortality,^26,27^ PPM causes residual left ventricle outflow obstruction, resulting in persistent left ventricle hypertrophy,^28^ impaired coronary flow reserve,^29^ and abnormalities of von Willebrand factor after AVR.^19^ Moderate or severe PVR, although infrequent, is an important limitation of TAVI that has been shown to be associated with increased risk of mortality and bleeding.^30,31^ Data on the association of aortic regurgitation with outcomes for noncardiac surgery are scarce; however, a single retrospective study^32^ among 167 patients with chronic moderate or severe aortic regurgitation found that chronic aortic regurgitation was associated with increased risk of cardiopulmonary complications (16.2% vs 5.4%; *P* = .003) and higher in-hospital mortality (9.0% vs 1.8%; *P* = .008). These results suggest that it is crucial to achieve optimal hemodynamic outcome when performing prophylactic TAVI before elective noncardiac surgery. If optimal device performance cannot be expected owing to challenging valve anatomy,^33^ surgical AVR may be prioritized as a prophylactic treatment. It remains to be investigated whether percutaneous or surgical reintervention for PPM or relevant PVR are associated with improve clinical outcomes for noncardiac surgery

### Limitations

The findings of our cohort study are exploratory and need to be interpreted in light of several limitations. First, the nature of this study did not allow us to obtain more detailed data on noncardiac surgery, such as anesthetic management, perioperative antithrombotic management, and perioperative complications (hemodynamic instability and worsening of heart failure), that are not systematically captured in the registry. Conversely, the robustness of the findings may be corroborated by the prospective data collection, completeness of follow-up, independent event adjudication, and rigorous statistical analysis by an independent statistical unit. Second, surgical procedures were heterogeneous, ranging from elective minor to urgent or emergent major operations, limiting the ability to make recommendations regarding specific surgical populations or clinical scenarios. However, risk of the 30-day composite end point was largely consistent regardless of surgical risk category or urgency of surgery. Third, given that echocardiography was not systematically performed at the time of noncardiac surgery, echocardiographic assessment of device performance was based on discharge echocardiography after TAVI. However, of note, no additional aortic valve intervention, such as postdilation procedure, valve-in-valve procedure, paravalvular leak closure, or surgical revision, was performed after discharge in the study cohort. Fourth, the cohort included only older patients who were offered and agreed to undergo noncardiac surgery; hence, the results may not be generalizable to younger patients or patients who were not offered or declined surgery. Perioperative mortality has been reported to be higher in patients aged 80 years and older, and the mortality rate in that study^34^ was similar to that of this cohort. Fifth, as with all observational studies, the possibility of residual confounding cannot be excluded, and the findings of the study are hypothesis generating.

## Conclusions

In this cohort study, suboptimal device performance, such as PPM and PVR, was associated with an increased risk of adverse outcomes after noncardiac surgery, while timing, urgency, and risk category of noncardiac surgery were not associated with increased risk. These findings suggest that noncardiac surgery may be performed early after successful TAVI. Further studies are needed to explore the optimal treatment strategy for patients with AS requiring noncardiac surgery.
